# Post-Burn Pruritus

**DOI:** 10.3390/ijms21113880

**Published:** 2020-05-29

**Authors:** Bo Young Chung, Han Bi Kim, Min Je Jung, Seok Young Kang, In-Suk Kwak, Chun Wook Park, Hye One Kim

**Affiliations:** 1 Department of Dermatology, Kangnam Sacred Heart Hospital, Hallym University, Seoul 07441, Korea; victoryby@naver.com (B.Y.C.); khmamy1029@naver.com (H.B.K.); luckyminja77@naver.com (M.J.J.); tjdjrdud@naver.com (S.Y.K.); dermap@hanmail.net (C.W.P.); 2 Department of Anesthesiology and Pain Medicine, Burn Center, Hangang Sacred Heart Hospital, Hallym University, Seoul 07247, Korea; 031132@hallym.or.kr

**Keywords:** burn, pruritus, neuropathy, sensitization, neuroleptic agent, TRPV, substance P, GABA, antidepressant, opioid antagonist

## Abstract

Post-burn pruritus is the pruritus that occurs after burn during the rehabilitation and healing process of burn wounds. The post-burn pruritus is a common and serious complication of burn injury, which severely lowers the quality of life of the patient. Many potential treatments are available for pruritus but there is no consensus of the best single treatment yet. The precise mechanism of post-burn pruritus has not been elucidated, but it appears to have pruritogenic and neuropathic aspects. Clinically, post-burn pruritus tends to be intractable to conventional treatment but rather responds to neuroleptic agents, such as gabapentin and pregabalin. During wound healing, various neuropeptides secreted from the nerves of the skin control epidermal and vascular proliferation and connective tissue cells. When keratinocytes are activated by an itch-inducing substance, they secrete a variety of inflammatory substances that increase the susceptibility of the itch receptor. There are two mechanisms underlying post-burn neuropathic pruritus. The first one is peripheral sensitization. The second one is the intact nociceptor hypothesis. An effective treatment for post-burn pruritus will also be effective in other neuropathic and intractable itching. In this review, we summarized the interaction and mechanism of keratinocytes, immune cells, and nerve fibers related to post-burn pruritus.

## 1. Status of Post-Burn Pruritus

Post-burn pruritus is the pruritus that occurs during the wound healing process after a burn. The onset of the pruritus may occur within a few days after burn and the prevalence of pruritus after burn is 80–100%, according to reports [1,2,3]. The prevalence of pruritus tends to decrease with time, but, in some cases, it has persisted for more than a few years and the prevalence rate has reached 40% even after 12 years [4]. Its risk factor appears to be higher in women, with larger burns, with more surgery, and with limb and facial burns [2,3]

There are many sensory discomforts including prickling, burning sensation, numbness, and stinging that occur in the post-burn state. Among the discomforts, post-burn pruritus is a very common and distressing problem affecting individuals afflicted by a burn injury. Itching has been shown to affect the quality of life of people with burns in aspects such as sleep disturbance, impairment of daily activities, and psychosocial well-being [3]. As a result, chronic itching has significant psychosocial repercussions. Specifically, the Dermatology Life Quality Index showed that the negative impact on quality of life and anxiety was greater for the group who experienced chronic itching (*p* < 0.001) [5]. Some studies show that the distress from pruritus can affect the quality of life and mental health and even induce suicidal ideas with high odds’ ratios (OR up to 1.4–3.1) [6,7]. Effectively alleviating the symptoms of post-burn pruritus is a major problem in the rehabilitation of all burn patients. However, although there are many potential treatments available for pruritus, there is not yet a consensus on the best single treatment.

## 2. Clinical Features of Post-Burn Pruritus

Acute post-burn pruritus occurs during the period from wound closure to the early remodeling phase of healing. The overall percentages of patients who suffer from mild to severe pruritus were 87%, 70%, and 67% at three months, one year, and two years post-burn, respectively [3].

The intensity of post-burn pruritus is usually severe (5 or higher on the visual analogue scale for pruritus) [2]. Post-burn pruritus is under-researched and many protocols reflect empirical approaches with limited evidence to support their effectiveness. However, it is hard to treat post-burn pruritus using conventional treatments such as topical corticosteroid [4].

The patients who suffer from post-burn pruritus have higher subjective scores for pain, stiffness, thickness, erythema index, and irregularity than those who do not. Among the histopathological differences, are that the burn scar has thicker epidermis, has thin and dense collagen bundles, has less elastic fiber, and has abundant mast cells [8,9]. 

In addition to the pruritus, a post-burn state can induce various sensory discomforts. Patients most commonly characterized their pain as a “pins and needles” sensation (46%). Other symptom reports included stabbing (13%), burning (13%), electric (7%), shooting (4%), and other pain sensations (32%) [10]. Post-burn pruritus may disturb sleep, interfere with social and daily activities, and may even worsen healing due to damage induced by intractable scratching [11].

## 3. Differential Diagnosis for Post-Burn Pruritus

When itching occurs in burn patients, the possibility of post-burn pruritus as well as other diseases needs to be considered. The diseases that require differentiation were classified into the three following categories.

### 3.1. Drug-Related Dermatologic Disorder

Patients take various medications, such as antibiotics, analgesics, and psychiatric drugs, in the process of burn treatment. In case of drug allergies to these drugs, it is usually accompanied by severe itching, which is difficult to differentiate from post-burn pruritus. There are some reported cases of drug eruption after a burn injury [11,12,13]. One of them was acute generalized exanthematous pustulosis (AGEP) and two were fixed drug eruption. Remarkably, the lesions of fixed drug eruption developed on the post-burn site. The interval between burn and drug eruption varied from two months to 22 years [11,12,13]. Therefore, physicians need to look closely at the drug history in relation to the possibility of drug eruption. The most important examination is to check if itching is localized on the burn site or generalized to the whole body.

### 3.2. Underlying Dermatologic Disorder

Several skin diseases have clinical features similar to those of burn injuries [14]. These include atopic dermatitis, psoriasis, and erythema multiforme. Some cases of these skin diseases are misdiagnosed as burn injuries. Patients who are unavailable for history taking, such as children, should be provided a process of differential diagnosis of underlying skin disease assumed to be post-burn pruritus. In addition, allergies, irritants, and moisturizers used in the treatment of burns and burn scars in patients with burns may cause allergic or irritant contact dermatitis that is pruritic. If erythematous patches or plaques exists over the burn or burn scar borders, the probability of contact dermatitis is high.

### 3.3. Other Sensory-Neural Disorders

Peripheral neuropathy is known to be the most frequent neurological complication of burns. The frequency of peripheral neuropathy after burns varies from 11% to 52%, depending on the study [15,16,17,18]. The neuropathy can easily remain undiagnosed because of the insidious onset [19]. Thus, post-burn pruritus should be distinguished from neuropathy with pruritus.

## 4. Mechanisms of Post-Burn Pruritus

### 4.1. Pathway and Mechanisms of General Pruritus

The keratinocytes contain various neurotransmitters and receptors like opioids, proteases, substance P (SP), nerve growth factor, neurotrophin 4, μ/κ-opioid receptors, proteinase activated receptor 2, tropomyosin-related kinase A, transient receptor potential vanilloid ion channels, and cannabinoid receptors 1 and 2 [20]. Peripheral nerve fibers, which are closely packed in the skin, sense environmental stimuli such as sensory perception, mechanical stimulation, and temperature. The somatic sensation is mediated by various types of primary sensory neurons present in the skin as free nerve endings. Approximately 5-20% of the total primary afferent C-fibers are activated by the itch-inducing substances secreted from cells other than neurons [21].

The nerve fibers of the skin that transmit the itch are unmyelinated, histamine-sensitive, C nerve fibers with a small diameter and a slow delivery rate, and the transduction media are mainly SP and calcitonin gene-related peptide (CGRP) [22]. The pruritic signal passes mainly through pruritus-selective unmyelinated C fibers that originate in the skin. They form a synapse with secondary neurons that cross to the contralateral spinothalamic tract and ascend to the ventromedial and dorsomedial nuclei of the thalamus, then provide the desire to scratch [23].

### 4.2. Pathophysiology for Neuropathic Pruritus

**Figure 1:**
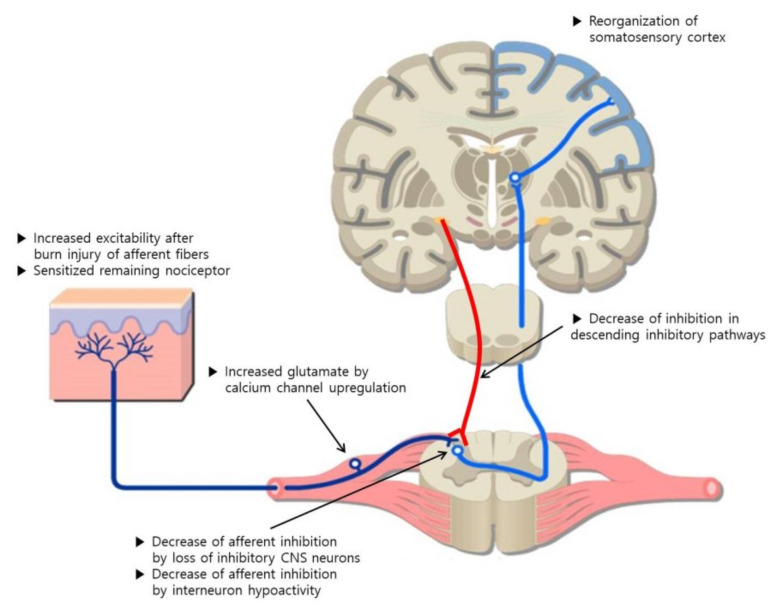
Mechanisms underlying post-burn neuropathic pruritus. Adapted from [25].

Neuropathic itch could arise as a consequence of a lesion affected by peripheral or central neurological disorders associated with tissue damage. Depending on the cause, neuropathic itch can be classified as peripheral or central. The mechanism of itch generation in neuropathic itch is unclear. There are some hypotheses that draw on the knowledge of neuropathic pain (Figure 1) [24,25]. There are two mechanisms underlying neuropathic pruritus, and the first one is peripheral sensitization. This means that injury to the peripheral afferent nerves results in altered input to the central nerve system (CNS).

To support the first mechanism, two main theories have been proposed. The first theory is the injured afferent hypothesis. Injury to peripheral afferent fibers causes neuromas, which consist of unmyelinated C-fibers growing from damaged axons. These neuromas have abnormal neuronal activity and excitability because of increased sensitivity. The second theory is the intact nociceptor hypothesis [25,26]. It is about nociceptors innervating the affected region after the injury. Normally, non-neuropathic itch can be caused by mechanical or chemical contacts that activate a few epidermal nerve endings with nociceptors [27]. These nociceptors become sensitized and develop spontaneous activity when injured by diseases. Such lesions cause changes at the molecular level including the release of chemical substances such as prostaglandins, bradykinin, and cytokines, and tumor necrosis factor-α, as well as receptors and ion channels. Because the C-fibers have the largest innervation territories, peripheral neuropathic itch can spread beyond the classic innervation area of damaged nerves. Proximal inflammation within damaged nerves and roots and the branching of C-fibers contribute to boundary enlargement [28]. 

The second mechanism underlying neuropathic pruritus is central sensitization. This means that autonomous, ongoing, aberrant activity develops in the CNS. Nerve injury can increase the activity of dorsal horn projection neurons. Peripheral injury induces upregulation of the α2δ subunit of calcium channels in the dorsal root ganglion and spinal cord with resulting increase in the release of the excitatory neurotransmitter glutamate. Injury can also cause the loss of afferent inhibition. Loss of A-beta (Aβ) myelinated fiber input due to injury promotes hypoactivity of interneurons that inhibit nociceptive afferents. Moreover, it can cause the loss of CNS inhibitory neurons and descending inhibitory neurons. The applicable CNS neurons are dorsal horn nerve fibers including Gamma-aminobutyric acid (GABA) interneurons.

### 4.3. Pathophysiology for Post-Burn Pruritus 

The precise mechanism of post-burn pruritus has not been elucidated, but it appears to have pruritogenic and neuropathic aspects (Table 1) [29]. However, it is believed that the neuropathic aspect is more predominantly involved in post-burn pruritus. Clinically, post-burn pruritus tends to be intractable to conventional treatments for pruritogenic pruritus such as antihistamines or topical and systemic corticosteroid. Rather, it responds to neuroleptic agents such as gabapentin and pregabalin. Because paresthesia often remains in the post-burn state (such as pins and needles, stabbing, or burning), there is also support for the notion that there may be a neuropathic component to post-burn pruritus [25]. This means that neural sensitization, as mentioned above, is the mainstay mechanism of chronic pruritus. Neuronal terminal loss, which is characteristic of neuropathic pruritus, can be found in post-burn pruritus [30]. 

After an injury, the skin-healing process involves three phases: An inflammatory phase, a proliferative phase, and a remodeling phase. During wound healing, various neuropeptides secreted from the nerves of the skin affect epidermal and dermal proliferation and inflammation [31,32,33]. When keratinocytes are activated by an itch-inducing substance, they secrete a variety of inflammatory substances that increase the susceptibility of the itch receptor. Histamine can increase keratinocyte production of inflammatory agents such as interleukin-6 (IL-6), IL-8, and C-C motif chemokine ligand 5 (CCL5) [34]. Similarly, treatment of keratinocytes with substance P and CGRP increases IL-1α and IL-8 expression [35].

Some chemokines secreted from keratinocytes directly activate neurons, while others work indirectly by stimulating other immune cells such as T-cells and mast cells to produce cytokines. These activate sensory neurons, such as histamine and IL-31, the latter of which not only directly activates cultured neurons but also causes severe scratching reactions without the involvement of mast cells when injected into animals [36]. Histamine acts directly on sensory neurons through histamine 1 (H1) receptors [37]. Mast cells are increased in post-burn hypertrophic scars, and the thicker the scars, the greater the itching [38].

Thymic stromal lymphopoietin (TSLP) from keratinocytes also can directly stimulate the nerve fibers [39], in which process the transient receptor potential vanilloid (TRPV) ion channel is involved. TSLP is secreted due to intracellular calcium ion increase after transient receptor potential vanilloid (TRPV) 1, TRPV3, or protease-activated receptor 2 (PAR2) activation in keratinocytes [40,41]. Increasing intracellular calcium triggers sequential activation of calcineurin and nuclear factor of activated T-cells (NFAT), and releases TSLP extracellularly [40]. In addition to direct action on neurons, TSLP also induces inflammatory immune responses by mast cells and T-helper-2 cells, thereby increasing cytokine production and indirectly contributing to itching [42]. However, activation of immune response by TSLP plays an important role in allergic inflammation and induces chronic itching [43]. In cases of acute itching caused by TSLP, T-cells, B-cells, and mast cells do not seem to be involved. Immunohistochemistry studies have shown that the TRPV3 channel, interleukin-31, and its receptors are more elevated in the epidermis of burn scars than in healthy skin [8,44]. Moreover, TRPV3 activation of epidermal keratinocytes from pruritic burn scar produces TSLP. TRPV3, TSLP, and transient receptor potential vanilloid receptor (TSLPR) are also highly expressed in fibroblasts from burn scars. TSLP induction by TRPV3 activation induced fibrotic molecules in fibroblasts in our recent study. TRPV3 channel induces dermal fibrosis via the TRPV3/TSLP/ mothers against decapentaplegic homolog 2/3 (Smad2/3) pathways in dermal fibroblasts [45]. Therefore, the molecules or mechanisms inducing post-burn pruritus are also involved in burn scar hypertrophy, which is why hyperproliferative scars are more itchy.

After the acute phase of healing, histamine is known to be less important to the mechanism of pruritus. In the chronic phase, it converts to the neuropathic type of pruritus characterized by antihistamine-resistant wounds with sensitized CNS. Increased neuropeptides, such as nerve growth factor, substance P, and the upregulation of calcium channel in the spinal cord, cause CNS sensitization in chronic burns. Thus, the CNS activates autonomously, which shifts maintenance into a chronic phase. Finally, a burn injury can induce synaptic reorganization. The post-burn state has been linked with alterations to the functional topography in the primary somatosensory cortex. This induces a reorganization of the somatosensory cortex that produces a distorted mapping of the skin [25].

## 5. Measurement for Post-Burn Pruritus

There is no established tool for measuring the severity of itch in post-burn pruritus. Clinically, visual analog scale (VAS) and numerical rating scale (NRS) are widely used. However, the methods have the limitations that they only reflect the severity of itching [25]. On the other hand, the five dimensions (5-D) itch scale [47] and the Leuven Itch Scale [48] can be used to measure not only the severity of itching but also various aspects of the itch.

The 5-D itch scale is a questionnaire grouped into five domains: Duration, degree, direction, disability, and distribution [47]. There was an evaluation administered to 234 individuals with chronic pruritus including burn injuries (*n* = 51). The 5-D scale was administered at baseline and after a six-week follow-up period. The 5-D scale score was highly correlated with a visual analog score (*p* < 0.0001) and with changes detected in the disease over the six-week follow-up period (*p* < 0.0001). The score was also correlated with the patient’s response pattern with the exception that the score was lower than expected because the burn site was too limited.

The Leuven Itch Scale is an instrument that approaches itch as a symptom and measures all aspects of itch: Frequency, duration, severity, distress, consequences, and surface [48]. There was an evaluation administered to 150 patients with chronic pruritus including burn injuries (*n* = 46). It evaluated the validity, reliability, and responsiveness of the Leuven Itch Scale. There were some invalid data about the frequency of itching and satisfaction with treatment and floor effects on the consequences of itching in patients with burns. Except for these scores, the Leuven Itch Scale proved to be a useful instrument for measuring pruritus.

## 6. Current Treatments for Post-Burn Pruritus 

**Figure 2:**
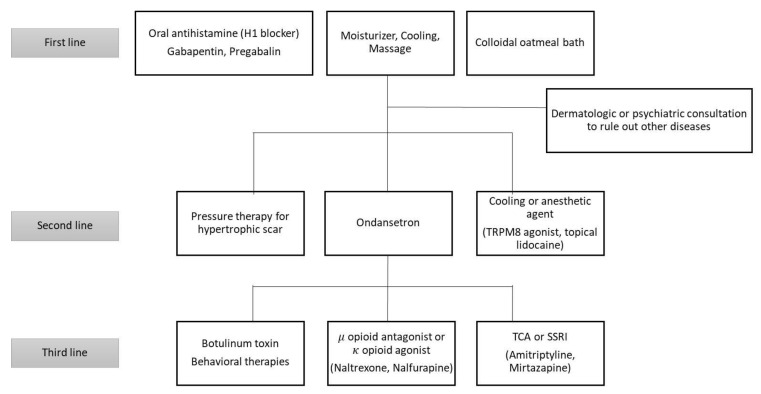
Algorithm of post-burn pruritus - modified treatment. Adapted from [49].

Various treatments are available for the relief of pruritis in patients with burns (Table 2). They range from antihistamines and topical emollients to psychological therapies, massage, and other dermatological treatments (Figure 2). However, there is no agreed upon and consistent management algorithm for this treatment [49]. Although the authors listed many of the treatments below, there is not enough experimental evidence for the treatments presented. Therefore, well-designed, randomized, placebo-controlled trials are still needed. Nevertheless, to tailor therapeutic regimens according to current evidence, patient values, risks, and resource considerations, the Grading of Recommendations, Assessment, Development, and Evaluation (GRADE) classification was made [1]. Twenty-three studies about therapeutic agents used on burns were analyzed by a multidisciplinary panel following the GRADE classification to rate individual agents. As a result of this multidisciplinary approach employing the GRADE classification, the antihistamines and gabapentin were recommended as the first-line pharmacological agents. Ondansetron was the second-line medication mentioned in this protocol.

### 6.1. Topical Treatments

The most basic and widely used topical treatments are topical emollients. The use of a moisturizer, a blend of physiological skin lipids made with the proper composition of the skin’s physiological lipids, ceramides, cholesterol, and fatty acids, is the basis for the treatment of itching. This is because it softens the stratum corneum and restores its barrier function, thereby relieving itching [50]. In addition, cooling of lesions and cooling agents are widely used, but there are no well-established studies to support them [1]. However, this is an integral part of the skin care routine, and the positive clinical experience allows their inclusion. There is a small pilot study assessing the effectiveness of topical dapsone with patients. The report on this study indicated a significant reduction in their symptoms. [51]. In one study, colloidal oatmeal was shown to reduce itch better than antihistamine preparations when used as a shower/bathing agent between the fifth and seventh day post-burn [52]. This may be related to the formation of an occlusive barrier on the skin and enhancing the maintenance of skin hydration and pH [53]. The itch and flare reactions associated with histamine release are reduced by pretreatment with lidocaine [2]. A study using a eutectic mixture of local anesthetics (EMLA) on healed but pruritic burn lesions showed a decrease in the number of pruritic episodes and antihistamine breakthrough doses compared with controls [54]. Because of their systemic toxicity, this treatment was limited to small areas of affected, fully healed skin. Topical 5–10% ketamine, in combination with 5% lidocaine and 5% amitriptyline, is an effective treatment for neuropathic itch [55]. A combination of topical ketamine-lidocaine-amitriptyline could be an option in post-burn patients for reducing the firing of defective nerves [5]. Although doxepin is a tricyclic antidepressant (TCA), it has highly potent histamine receptor-blocking properties [56]. Topical doxepin cream has been used for pruritus of various dermatological disorders. There is a study comparing topical 5% doxepin cream with standard therapy (oral antihistamines, skin moisturizers, and sedatives) on healed pruritic wounds. It was found that pruritus stopped in 55% of patients compared with 10% in the control group [57]. This treatment is recommended for use on fully healed and epithelialized pruritic burn wounds not exceeding 20% total body surface area (TBSA). Topical steroid with tretinoin has been reported to reduce scar formation in the early stages of burn [58]. Topical steroid can help burn scar to recover earlier without an anti-inflammatory effect. Low-dose topical steroid (1% hydrocortisone) also has anti-pruritic effect in selected people without side effects [59].

### 6.2. Systemic Treatment

#### 6.2.1. Antihistamine

The mainstay of therapy for post-burn pruritus is still antihistamines. However, they may be useless for some patients in the chronic stage [60]. First-generation antihistamines bind to histaminic, muscarinic, alpha adrenergic, and serotonergic receptors, whereas second-generation compounds affect mainly non-histamine receptors. The latter causes a side effect of sedation, resulting in an antipruritic effect [61]. The efficacy of second-generation antihistamines in reducing pruritus is lower than that of the first-generation ones [62]. However, even the first-generation antihistamines had minor effect for relieving post-burn pruritus and was effective in only 20% of patients [2]. Another study suggested that the combination of cetirizine and cimetidine provided better outcomes in numerical itch scores when compared with diphenhydramine and a placebo [63]. Loratadine has been found to be an effective alternative drug in pediatric burn patients who are refractory to diphenhydramine and/or hydroxyzine therapy [64].

#### 6.2.2. Opioid Receptor Agonists or Antagonists

There are three types of opioid receptors (mu, kappa, and delta). All of them increase the density of keratinocytes and fibroblasts in hypertrophic scars. Regarding pruritus, ligand binding to mu opioid receptors induces pruritus, while ligand binding to kappa opioid receptors inhibits pruritus. There are 15 cases in which, after administration of naltrexone (mu opioid antagonist), it was reported that symptom relief was better than with antihistamine [65,66]. Although kappa-opioid agonists show success in the treatment of pruritus with other etiologies, this has not been proven for post-burn pruritus.

#### 6.2.3. Ondansetron

C-fibers conducting itch sensations are known to be stimulated by serotonin [67]. Therefore, serotonin or 5-hydroxytryptamine (5-HT) is related to the pathophysiology of pruritus in various disorders. In a double-blinded, randomized, crossover trial, ondansetron (5-HT receptor blocker) was shown to yield greater effect than with antihistamine [68].

#### 6.2.4. Gabapentin and Pregabalin

Gabapentin is another agent widely used for neuropathic pain or pruritus. It inhibits the release of the excitatory neurotransmitters, like glutamate, on the voltage-gated calcium ion channels in the dorsal horn of the spinal cord [69]. Moreover, inhibitory neurotransmitters, like Gamma-aminobutyric acid (GABA), are increased by alteration of the activity of glutamic acid decarboxylase in neurologic tissues. Gabapentin also decreases the release of CGRP and SP from primary afferent neurons [69,70]. It showed clinical improvement on pruritic burn wounds. One study even showed better symptomatic benefits than with second-generation antihistamine cetirizine [71,72]. For patients with post-burn pruritus, the dose of gabapentin was 5–10 mg/kg dose in pediatric patients and 300–900 mg/day in divided doses in adults [71,72]. In particular, for patients with chronic itching post-burn, gabapentin monotherapy resulted in better outcomes than with antihistamine because of the ineffectiveness of the latter [61].

Pregabalin, an analog of gabapentin with more potency and longer half-life, also has benefits for treatment of neuropathic itch and was found to be superior to antihistamines like cetirizine and pheniramine maleate [70,73,74]. The treatment involves twice-daily dosing. In a double-blind, randomized, and placebo-controlled study, mild or moderate itch of patients was resolved better by pregabalin or by a combination of pregabalin and antihistamines [74]. For refractory post-burn itch, the combination of gabapentin and pregabalin can be used. In a study of burn patients, all patients had itching better relieved by combination therapy than by monotherapy [73].

#### 6.2.5. Antidepressants

Even though there is no sufficient data regarding the effect of antidepressants in post-burn pruritus, antidepressants have been used for various pruritus according to the various mechanisms of action.

Tricyclic antidepressants (TCA), such as doxepin and amitriptyline, reduce itching as an antagonist of histaminergic and cholinergic receptors. However, it is not recommended due to the uncertain effect and possibility of severe side effects [75]. Selective serotonin reuptake inhibitor (SSRI), paroxetine, increases serotonin by suppressing serotonin reuptake in nerve synapses. By this effect, it can be used for refractory pruritus and has been effective in some cases [76]. Although there is still a lack of data on post-burn pruritus, it can be used in people with psychiatric complications such as depression and anxiety. Selective serotonin and norepinephrine reuptake inhibitor (SNRI), mirtazapine, has been reported to effect nocturnal pruritus accompanied with anxiety and depressive symptoms [77]. Mirtazapine is also characterized as a safe drug with no serious side effects.

### 6.3. Extracorporeal Shockwave Therapy (ESWT)

ESWT is using acoustic wave to elicit protein response via mechanotransduction. In burn-related pruritus, a group performed three, focused ESWT sessions with 0.05–0.2 millijoule (mJ)/mm^2^ and 2000 shots per treatment. Pruritus could be significantly reduced as early as after 14 days. The authors hypothesized that focused ESWT might work anti-inflammatorily in this regard. Even an interaction with the numerous cytokines as well as with CGRP appears feasible [78,79].

### 6.4. Physical Treatment

There are physical treatments for post-burn pruritus, such as pressure therapy and massage therapy. Studies have indicated that compression can be effective against burn pruritus accompanying active hypertrophic scars [80,81]. The pressure may control collagen synthesis by limiting the supply of oxygen and nutrients, thereby blocking the conversion of fibroblasts into myoblasts [82]. In a study on burn patients with scar tissue areas of moderate size, patients receiving massage twice weekly showed improvement during the whole study period [83]. The massage helps to moisturize the skin layer and increase vagal activity, thereby reducing stress hormones circulating in treated patients [84].

### 6.5. Other Treatments

Treatment of pruritus by botulinum toxin injection was also reported. It may act in neuropathic itch [85,86]. This is because botulinum acts on TRPV1 present in C-fiber by diminishing neuropeptides [87,88,89]. Evaluation regarding the efficacy of botulinum toxin in patients complaining about post-burn pruritus was reported [90]. After four weeks of botulinum toxin injection, the patients did not have any itching sensation, even in severe patients in the past. During the follow-up, the mean symptom-free duration was about nine months.

Steroid injections can be used in limited post-burn pruritus with scars. Intralesional triamcinolone injection has been reported effective in post-burn keloid scar [91]. Intralesional injection not only reduces pruritus, but it also reduces the size, color, and texture of keloid scar to be normal. Although there were side effects, such as skin contractions, changes in pigmentation, and pain, intralesional injection could be considered in small scars.

## 7. Conclusions

Post-burn pruritus is a serious complication of burn injury that severely lowers the quality of life for the patient. The pathophysiology is not fully understood and an effective treatment has not yet been developed. An effective treatment for post-burn pruritus will also be effective for other neuropathic and intractable itching, and vice versa.

## Figures and Tables

**Table 1:** Cells and molecules involved in the pathophysiology for post-burn pruritus.

Cells and Molecules		Mechanism
Acute phase	Keratinocyte	Keratinocyte secretes a variety of inflammatory substances which induce pruritus; histamine, calcitonin gene related peptides (CGRP), Substance P, etc. [20]
	Mast cell	Mast cell releases histamine into post-burn lesion [38]
	Histamine	Histamine appears to be a key initiator of impulses predominantly in the initial stages of healing after burn, but the role of histamine in post-burn itch is minimal [46] It helps keratinocyte produce inflammatory agents [34,37]
	Substance P Calcitonin gene related peptides (CGRP)	Substance P and CGRP are the main transduction medias involved in C fiber. Substance P and CGRP increase IL-1α, IL-8, and TNF-α mRNA expression [22,35]
	Transient receptor Potential vanilloid (TRPV) 1,3	TRPV 1 and 3 increase intracellular calcium, triggering activation of calcineurin and NFAT, resulting in a release of TSLP extracellularly [40,41]
	Thymic stromal lymphopoietin (TSLP)	TSLP activate sensory neurons directly to cause an itch. TSLP induce inflammatory immune response to mast cells and T helper 2 cells [39,42]
	Interleukin-31 (IL-31)	IL-31 stimulates the afferent neurons which have TRPV1/TRPA1. IL-31 amplify the inflammation of the skin through chemokine induction, causing T-cell recruitment [36]
Chronic phase	Nerve growth factor Substance P Histamine Neurokinin A Eicosanoids Bradykinin	These inflammatory substances cause CNS sensitization in chronic burns by upregulating C-fiber activation [25]
	Gamma-aminobutyric acid (GABA)	Degeneration of GABA interneurons decrease inhibition to nociceptive pathway and contribute to hypersensitivity [25]
	TRPA1 TRPV4 TRPV3 IL-31	Their receptors elevated more in the burn scar resulting in an increase of TSLP [8,44]
	Norepinephrine 5-Hydroxytryptamine(5-HT) Serotonin Dopamine	These molecules exist in the descending pathway with anti-nociceptive activity. Disinhibition in this pathway results in excitability in the CNS [25]

**Table 2:** Treatments for post-burn pruritus.

Type	Treatment	Mechanism	Reference	Methods	Main results
Topical	Moisturizer & Cooling agents	Softening the stratum corneum and restoring the barrier function	No established study	No established study	No established study
	Dapsone	Anti-inflammatory effects	Bauling et al. [51]	Observational study (*n* = 8) Topically administered for 14 days	Significant relief of itch to 5 patients
	Colloidal oatmeal	Formation of an occlusive barrier and maintenance of hydration and pH	Matheson et al. [52]	Cohort study (study 17, control 17) 5% colloidal oatmeal + liquid paraffin vs liquid paraffin bath and moisturizer	Decrease in itch and antihistamine usage in the colloidal oatmeal group
	Eutectic mixture of local anesthetics (EMLA)	Analgesic effects	Kopecky et al. [54]	Observational study (*n* = 5) EMLA is applied for 1 h or 2 h.	Decreased mean number of pruritic episodes and needed medication
	Doxepin	High potent blocking properties to histamine receptor	Demling et al. [58]	Case-control study (*n* = 41) Doxepin + moisturizer vs moisturizer + antihistamine	Significant reduction in itch and erythema
Systemic	Antihistamine	Reducing the effect of histamine	Baker et al. [63]	Randomized double-blinded, placebo controlled study (*n* = 32) Cetirizine + cimetidine vs Diphenhydramine + placebo	Improvement and moderate impact in cetirizine + cimetidine group compared to diphenhydramine + placebo group
	Opioid	Not proved yet.	Jung et al. [66]	Case series (*n* = 15) Naltrexone was applied to all patients for 2 weeks	Decrease in the severity of itching
	Ondansetron	Decrease in the stimulation of C-fibers induced by serotonin	Gross et al. [68]	Randomized double-blinded study (*n* = 17) 4mg Ondansetron vs 25mg diphenhydramine	Better decrease in the severity of itching than antihistamine
	Gabapentin	Based on the similarity of neuropathic pain and itch	Mendham et al. [71]	Observational study (*n* = 35) Gabapentin was applied to patients 5mg/kg or above (max 10mg/kg/day).	Decrease in the need of antihistamine within 24 h.
Physical	Pressure therapy	Controlling the collagen synthesis by limiting the oxygen and nutrients	Leung et al. [81]	Observational study (*n* = 100) Pressure garment was applied for an average of 10 months.	The assessment of itch was satisfactory to most of patients.
	Massage therapy	Moisturizing skin, increasing vagal activity, and reducing circulating stress hormones	Field et al. [84]	Experimental study (study 10, control 10) Standard treatment + massage vs standard treatment	More decrease in pruritus, pain, and anxiety than control (*p* < 0.001)

## Abbreviations

AGEP	Acute generalized exanthematous pustulosis
SP	Substance P
CGRP	Calcitonin gene-related peptide
GABA	Gamma-aminobutyric acid
TRPV	Transient receptor potential vanilloid
GRADE	Grading of Recommendations, Assessment, Development, and Evaluation
EMLA	Eutectic mixture of local anesthetics
TCA	Tricyclic antidepressant
5-HT	5-Hydroxytryptamine
SSRI	Selective serotonin reuptake inhibitor
SNRI	Selective serotonin and norepinephrine reuptake inhibitor
